# Hospital and Institutional News

**Published:** 1914-03-07

**Authors:** 


### HOSPITAL AND INSTITUTIONAL NEWS.

### A SCHOOL FOR SECRETARIES.

Mr. William Rutter, assistant-secretary to the King Edward VII.'s Hospital, Cardiff, has just been appointed secretary of Chester General Infirmary. Mr. Rutter has only been at the Cardiff Hospital for about eleven months, but he went there from the North Staffordshire Infirmary, Stoke-on-Trent, with high testimonials and nearly eight years' experience of hospital work. It is interesting to note that within a little over three years no fewer than three assistant-secretaries have been promoted from the Cardiff Hospital—Mr. A. Ernest Wilkes having been appointed secretary of the St. Mary's Hospital for Women and Children, Plaistow, E.; Mr. Frank Inch secretary of the Walsall and District Hospital, Walsall; and now Mr. Rutter has been promoted to Chester. This is strong evidence that the King Edward VII.'s Hospital, Cardiff, is generally regarded as an excellent training school for those who are desirous of succeeding in the profession of hospital secretary; and, indeed, its records show that for extent and variety of work it holds a high place, and in some respects, notably in the method employed for obtaining payments from in- and out-patients, the experience is unique.

### RUBBER FLOORS FOR EDINBURGH ROYAL INFIRMARY.

An offer is announced to the Edinburgh Royal Infirmary of six hundred square yards of rubber flooring, for which the Rubber Growers' Association is responsible. A motion to accept the offer was made by Dr. Johnston, who remarked on its generosity, as he understood the cost of rubber floors to work out at 25s. per square yard. Needless to say, the offer was accepted, and institutional managers will soon have an opportunity of testing on a large scale the virtues of this hitherto prohibitively expensive material. In an article on the "Ward Floor," which appeared in The Hospital of April 6, 1912 (page 13), the following statement on rubber floors was made, and is worth requoting in this connection: "Lately, some hospitals have tentatively experimented with rubber tiles. These are very durable, clean, comfortable to the feet, and make excellent flooring. They have been laid down with very satisfactory results in several public buildings—notably in the White Star buildings at Liverpool—but they are still so expensive that their cost is almost prohibitive in a general ward."

### THE MARRIAGE OF MALE ATTENDANTS.

On April 1 next the improved conditions of service which the Mental Hospitals Committee of the London County Council has adopted for the male attendants in their institutions will come into force. Prominent among these is the right to marry, which is to be conceded to a greater number, after at least five years' service, with the correlative privilege of living outside the institution, for whom a money allowance in lieu of emoluments will be provided on the understanding that those living out will be required in rotation to sleep in for stated periods. The most important of the other improved conditions are the raising of the commencing salaries of head attendants from £53 to £60 a year, and of those for second class attendants from £31 for six probationary months to £35 on confirmation instead of to £33, though under the new scale the probationary period is increased to one year. The maximum salaries remain the same—namely, £80 for the head and £53 for the first class, except in the case of the second class, when the maximum is increased from £43 to £47. The new scale, which will affect some 1,080 members of the staff, is expected to cost an additional £1,150 during the next twelve months. We are quite sure that the money is well spent, and that the higher salaries and freedom to marry will react for the advantage alike of the attendants and the patients whom they serve.

### MR. C. H. WELLS AND GUY'S HOSPITAL.

The appointment of Mr. C. H. Wells as clerk to the governors of Guy's Hospital, in succession to the late Mr. Henry Williams, who died last December, is the occasion of an interesting *résumé*, in last Saturday's *Gazette*, of Mr. Wells' previous work at the institution. As the senior lay-post at Guy's, it comes to Mr. Wells after thirty-four years' work at the hospital, for he became clerk to the dean of the medical school in 1880. As secretary to the treasurer, which post he has held since its creation (which synchronised with Sir Cooper Perry's appointment as superintendent on relinquishing the post of dean of the medical school), Mr. Wells has always been concerned in finance, which perhaps presented an even more than ordinary difficult problem to Guy's during the last quarter of the nineteenth century. It was in a City bank that Mr. Wells acquired experience before coming to Guy's, and one of his first duties, additional to those in the office of the dean, was to become financial manager to the *Gazette*. The business side of the paper has remained under his control, and the large developments of the school, which followed Sir Cooper's appointment as dean a few years later, owe much to Mr. Wells' abilities. The foundation of the dental school, the building of the residential college, the reconstruction of the school "labs.," and the amalgamation of the various societies into the Students' Clubs Union were all features of this active period. The great work undertaken by Sir Cooper Perry on becoming superintendent, of retrieving the losses which agricultural depression had caused to the hospital, led to the issue of a great appeal, and with it the creation of a new post, that of secretary to the treasurer, which Mr. Wells has held till to-day. The appeal created a record, since no less than £166,000 was raised at the festival dinner, at which King Edward, then Prince of Wales, presided. With so long and varied an experience of Guy's as this, Mr.

Wells brings everything that can be brought to his new post. Mr. W. J. Curry has been appointed assistant clerk.

### THE BRADFORD INFIRMARY PROJECT.

An interesting statement on the sale of the Bradford Royal Infirmary is made in the latest annual report, which was presented to the governors last week. The sufficiency of voluntary effort to meet working expenses is insisted on, but the problem of raising enough money to rebuild the institution is also emphasised. In addition to the £100,000 to be provided by the Corporation, the public is asked to raise the £80,000 already promised to a similar amount, and the charge of extravagance in regard to this sum total is dealt with. "If," says the report, "the scheme be carried to its completion in the future, the hospital will contain about the same number of beds as exist at Leeds General Infirmary, and nearly the same as at Manchester Royal Infirmary. The cost to Bradford is estimated at about one-half of the sums spent by those two cities upon their infirmaries. The scheme which has been adopted is of a comprehensive character, and covers far more than the immediate requirements." Mr. J. Priestman assured the meeting that there would be no difficulty in supporting the new institution by voluntary effort. This view seemed to be shared by other speakers, most of whom characterised the proposal as one of common sense, and the only implied criticism was that it should have been completed before this. The real test of its support in Bradford will be the ease or difficulty with which the additional £20,000 will be raised.

### GERMAN AND AMERICAN CONSTRUCTION.

We have received a reprint from *The Hospital World* of Canada of a paper by Dr. John Brown, the secretary of the American Hospital Association and superintendent of the Detroit General Hospital, which contains several blocks relating to various hospitals. Its aim appears to be to make a comparison between German and American construction, but the practical value of the paper is neutralised by the omission of the names of the hospitals and the buildings to which the illustrations relate, the absence of any scale or measurements, and of necessary practical details. Would it not be possible for Dr. Brown, with the aid of the architect, Mr. Stratton, of the Detroit General Hospital, to go through his paper, to supply the omissions referred to, and then to reprint the paper and circulate it? As a practical illustration we quote the following sentence from page 9 of the reprint: "The ventilating apparatus of the lavatories, kitchen and sink rooms is made particularly effective in order quickly to carry off the vapours and malodours which form there." Such a statement is not illuminating, and could not aid the most practical and able experts in construction.

### A POOR-LAW COLONY FOR SANE EPILEPTICS.

In last week's number of The Hospital we drew attention to the lack of suitable provision by Boards of Guardians for epileptics and the feeble-minded. A scheme which has just received the sanction of the Local Government Board shows that this reproach will no longer hold good so far as some of the Unions in the North of England are concerned. Twenty-four Unions in the counties of Cumberland, Durham, Northumberland, and Yorkshire, including the Unions of Auckland, Carlisle, Darlington, Durham, Hartlepool, Lanchester, Middlesbrough, Newcastle, South Shields, Sunderland, and Tynemouth, have combined to establish a colony in which are to be received epileptics and feeble-minded, whether certifiable or not. The joint committee of these Unions has bought Prudhoe Hall and estate, beautifully situated in a secluded portion of the Tyne Valley. The Hall stands in about 130 acres of ground, and an additional 100 acres adjoining the estate are available and may be purchased. The Hall is a large mansion, and the committee state it will afford accommodation for about 80 cases at once, and that other buildings can be added when necessary. As the population of the combined Unions exceeds two millions it is obvious that the colony will finally assume very large proportions. For this, however, the joint committee appears to be prepared, as in the preliminary negotiations, when only 16 Unions had accepted the idea of combination, it was stated that an institution with about 400 beds would be required.

### A RISK AT THE START.

Children and adults of both sexes are to be received into the colony, so that its successful administration will be a difficult and complicated matter. Success will depend more than anything upon a skilled and adequate staff, and especially upon the selection of a suitable medical superintendent. He will have to be a man of exceptional administrative ability and experience, with a thorough and practical knowledge of epilepsy and feeble-mindedness and the treatment of these conditions. The danger we fear is that, starting as is proposed in a small way, the committee will not realise how much the success of the scheme will depend upon the selection of the chief medical officer, and may assign to the post remuneration and conditions which will fail to secure the best candidates available.

### A MENTAL HOSPITAL EXPERIMENT.

An interesting experiment in connection with the After-Care Association for poor persons discharged from mental hospitals is referred to in the annual report of the Association. Since last November an attempt was made in connection with some of the southern mental hospitals to receive on trial certain patients who had been discharged. The satisfactory results achieved so far are encouraging enough for the extension of similar help to other mental hospitals to be hoped for. In every branch of institutional work the problem of after-care is receiving increased attention, and the endeavour to set on their feet again patients discharged from mental hospitals is one that will be watched with interest, for such patients are popularly regarded as even more complete social derelicts than others. The work, moreover, is one which, being still in its experimental stage, is eminently suited to the enterprise of a voluntary body.

### THE LATE LORD STRATHCONA AND FORRES HOSPITAL.

Ex-Provost Lawrence, chairman of the Leanchoil Hospital, Forres, N.B., in announcing the text of the late Lord Strathcona's bequest of £10,000 to the hospital, which was noted in The Hospital as soon as his will was made known, stated that this sum brought up his gifts to the hospital to the magnificent total of £18,300. When it is remembered that the hospital possesses only twenty beds, the amount of its debt to the late Lord Strathcona and of his interest in it can be well appreciated. The hospital has also lost a very valued friend in Lady Macpherson-Grant, the wife of the hospital's president, who was the second person to sign the visitor's book after the hospital was opened twenty-five years ago, and who was constant in her enthusiasm till the day of her death.

### SIR BLAND SUTTON AND MIDDLESEX HOSPITAL.

The hitherto anonymous donor of £15,000 for the building of a new pathological block and institute of hygiene in connection with Middlesex Hospital revealed his name at the annual court last week, when Prince Alexander of Teck read a letter from Sir John Bland Sutton explaining his position in regard to his gift. "I preferred to remain anonymous for a season," he wrote, "as it would be my lot to take part in the organisation of the institute, and I did not wish to have any undue influence in this matter. Now that my colleagues have agreed on the chief points in this organisation there is no longer any need for anonymity." Sir John Sutton then expresses the hope that someone will come forward to endow the directorship of the institute, which needs a considerable sum for its annual upkeep. If we may judge by the increase in subscriptions which Middlesex Hospital received last year, and by its good fortune in the matter of legacies, there seems a reasonable prospect of this hope being realised. The new institute will increase the hospital's resources as a teaching centre by making diagnosis a more exact science. We hope, at any rate, that the present donor's generous example will quickly find an imitator, especially in the direction indicated by his letter.

### NORTHAMPTON HOSPITAL AND THE "LETTER" SYSTEM.

The recent report of Sir Henry Burdett upon the Northampton General Hospital may, it is hoped, serve to call attention to the efforts which have lately been made to arouse public interest in Northampton on the subject of the abuses of the "letter" system. The movement has been initiated by Mr. Montague Browne, a governor of some forty years' standing. In a letter to the *Northampton Daily Chronicle* he has related the difficulties which persons in need of treatment, and suitable recipients of the hospital's charity, must sometimes face in order to secure the necessary passport. His plea has been supported by several correspondents. It is urged also that the appointment of an almoner would not only secure that suitable patients should be assisted, but that they should be enabled to take full advantage of the medical treatment received. It appears that at Northampton grants made by public and other bodies to the hospital have involved, to a quite unusual degree, much bargaining as to the number of "letters" which should be received, proceedings unworthy of a charity and its supporters, but apparently inevitable under the present system. On another page in this issue will be found a letter from the Chairman of the Board of Management containing comments on Sir Henry's Report.

### DR. CHAPPLE ATTACKS THE "LONDON."

The cause of registration has often been singularly unfortunate in its advocates, and Dr. Chapple's introduction of the latest Registration Bill in the House of Commons on Tuesday was no exception. He explained that the Bill, without compulsion of cost to the State, set up a Nursing Council to prescribe a minimum standard of training, to grant certificates to those who passed its examination, and to keep a register. Of 200 institutions lately circularised, he said that ninety-nine declared themselves very short of nurses, the quality of applicants was lower, and the public at present imposed upon. Massage houses, private hospitals, and quack institutions issued certificates; and the uniform was abused for wrongful purposes. Dr. Chapple passed from these arguments to attack the London Hospital. He said it was the only great hospital which opposed registration, and that it exploited nurses for its financial advantage. At this point Sir G. Younger interrupted with the remark: "That is a very serious charge." "I know, and shall substantiate it," retorted Dr. Chapple.

### A SCENE IN THE HOUSE OF COMMONS.

Sir G. Baring thereupon asked the Speaker whether the hon. member, in introducing a Bill under the ten minutes' rule, was entitled to attack an institution. The Speaker's reply was that the proper course was to state the clauses of the Bill, and not to attack institutions which had no representatives to defend them. Dr. Chapple, however, continued in the same vein, saying if a hospital only gave a two years' training, and in the third year sent out a nurse to whom it paid only 11s. 6d. a week when it charged two guineas—when the Speaker pulled him up. Dr. Chapple then said that a Select Committee had reported in favour of registration, and that the Medical Council and the British Medical Association were in favour of it. Mr. Booth opposed the Bill, saying that the introducer had overstated his case, and exaggerated the abuse of the uniform. On pressing the motion to a division, there voted for leave to introduce the Bill 311, and against 82; the majority in favour being 229. The Bill was then read a first time, but the net result of Dr. Chapple's tactics is to have created opposition at a Parliamentary stage where there is usually none—a result on which registrationists will hardly congratulate him.

### THE LISTER MEMORIAL.

The combined scheme for an International Memorial to Lord Lister does not seem to be making very rapid progress, to say the least. It is therefore satisfactory to learn that the æsthetic portion of the proposals has got as far as the commissioning of Sir Thomas Brock to execute the medallion portrait which is to be placed in the Abbey. The more ambitious plan—namely, for the establishment of an international fund for the advancement of surgery—is still in serious need of subscriptions. As a matter of fact, we think that the Memorial Committee made a mistake in casting their net too wide, for an international charity is apt to be one in which no one takes the smallest interest; and though they were right to welcome subscriptions from every quarter, Lister, after all, was an Englishman, his work was done in the United Kingdom, and his fellow-countrymen should have the chief responsibility in the memorial which they have instituted. Had less been made of its international character the claims of the fund might have been better advertised at home. The public memory is as short as the life of its daily newspapers, and no reputation, however great, can survive delay in its appeal for a public memorial.

### AN INSOLVENT INSURANCE COMMITTEE.

When establishing municipal tuberculosis dispensaries the Metropolitan Borough Councils were assured that the cost of treating insured persons would be repaid to them by the Insurance Committee for the County of London. The promise has not been kept, with the result that the Councils have been compelled to accept about half the sum they have spent on the treatment of insured persons. The funds at the disposal of the Insurance Committee have been inadequate to meet the cost of treating insured persons, and the Committee is now stated to be in an insolvent position. The Poplar Borough Council have declined to treat any more insured persons at their dispensary, and the Lambeth Borough Council have adopted a resolution that they are prepared to continue the treatment of insured persons providing the Insurance Committee will enter into an agreement to pay the Council the cost of such treatment. Dr. MacKeith, who is a member of the Council, hoped they would fix a day when they would cease to treat insured persons at the dispensary unless the Insurance Committee agreed to repay them for the cost of the treatment. He would oppose the treatment of insured persons at the dispensary at the expense of the rates. He pointed out that the panel doctors were paid sixpence for each person on their lists in respect of tuberculosis treatment, yet they were referring these patients to the dispensaries for treatment. The sixpences should not go to the panel doctors, who had no right to take the money and send the patients to the dispensaries for treatment. The money should come to the Council. The matter, we understand, is now engaging the attention of the Insurance Commissioners.

### LYING-IN HOSPITALS AND THE INSURANCE ACT

One noteworthy effect of the Insurance Act upon maternity hospitals has come home to the City of London Lying-in Hospital during the past year. The number of women who are receiving maternity benefit and being attended by midwives has led the authorities to limit the work of the outdoor department to the home district. There is a good deal to be said in favour of this limitation in any case, provided that those who require treatment suffer no hardship from it, and it would seem, therefore, that maternity benefit is providing midwifery treatment close at hand for those who have had previously to rely upon the outdoor work of lying-in hospitals. This should make the hospital's work more easily controlled, and apparently shows that more women are being satisfactorily provided for.

### PRISONS AS SANATORIA.

The Tuberculosis Exhibition at Preston which Sir James Barr opened last Monday provided him with an opportunity, of which he was not slow to make use, to drive home the lesson of the high death-rate now found in sparsely populated districts, which seems to show that something more than the tubercle bacillus must be reckoned with in stamping out the disease. For instance, he pointed out that prisons were about the healthiest sanatoria in the country, their death-rate being lower than that of the House of Commons which legislates for them, in spite of the fact that institutions, like the forecastle of ships, used to be recognised centres of infection. To-day a prolonged stay in one of the prisons would probably lengthen life. Since 1871 the fall in the tuberculosis death-rate was greater than that of other diseases, which led one to ask if the high mortality of the towns in the 'sixties had cut off the susceptible and rendered the population more or less immune. Here is an opportunity for tuberculosis officers and sanatorium experts to follow up Sir James Barr's contention, and to explain what other factors besides the specific bacillus should engage the attention of the anti-phthisis campaign.

### A MODEL SOCIETY.

The Leicester Saturday Hospital Society, which contributes approximately two-fifths of the income of the Leicester Royal Infirmary, is to be congratulated on another record year. The total receipts are £15,633, an increase of £500 on the previous year. A year's working of the National Insurance Act has shown that the work-people are still enthusiastic in their support of the Society. In the two convalescent homes of the Society 1,738 patients were received during the year, an increase of 383 on the previous year. The average cost of maintenance per week has been 15s. 6d. per patient and member of the staff. At the recent annual meeting of the Society, held at the Royal Infirmary, Sir Edward Wood was re-elected president, Mr. S. A. Gimson and Mr. J. Gipson Clarke vice-presidents, and Mr. J. A. Corah treasurer. By the authority of the High Court, a sum of £2,695, the balance of the "Higgs" bequest for the maintenance and endowment of a convalescent home, has been invested in the names of the Official Trustees of Charitable Funds towards the annual income of the Swithland Home, which is the Women's Home of the Society. While this is a model for the Bristol fund to aim at on the one hand, the precise means of labour representation should also be quoted by labour men in Bristol who give prominence to the Leicester Society's financial results.

### A HOSPITAL THREATENED WITH BANKRUPTCY.

For some time now, as has been noted periodically in these columns, financial adversity has been overtaking the Shadwell Children's Hospital. At the annual court of governors, just held, under the chairmanship of Colonel Charles Needham, a truly dismal story had to be unfolded. The report of the committee of management stated that the fortunes of the charity had never been at so low an ebb. Donations fell from £9,458 to £6,081, legacies from £1,950 to £50, and annual subscriptions from £1,717 to £1,576. At the same time expenditure increased. The Chairman expressed the belief that nine out of ten people in London do not know where the hospital is, and that they ought to advertise themselves a bit more. Certainly something must be done, effectively and quickly, if this hospital is not to close its wards and its doors in bankruptcy. While crediting Colonel Needham and his fellow-committeemen with every desire to amend the fortunes of the institution, it is yet obvious that the management is not exactly a success, and that it is capable of very great improvement. We think the governors should take in hand very seriously the question of finance, and do their best to get some new blood imported into the committee if the affairs of the hospital are not to go from bad to worse.

### CYPRUS AS A HEALTH RESORT.

On Friday last week, at a meeting of the Epidemiological Section of the Royal Society of Medicine, Sir Ronald Ross, K.C.B., F.R.S., lectured on the subject of his investigations in Cyprus and Greece regarding the prevalence of malaria. Cyprus, although practically a British possession, is not a place about which much is heard nowadays. At one time it was on the way to become a sort of health resort for Egyptian officers and their families. Its proximity and its pleasant climate made the island a convenient substitute for the home country during the hot season, but after a few years it lost its vogue. Sir Ronald, as is well known, has succeeded in showing how comparatively easy it is to stamp out malaria in various localities by abolishing shallow pools and streams, which form the breeding places for the parasite-bearing mosquitoes. In spite of the fact that malaria in Cyprus is only found here and there in the island, administrative measures have so far not been brought to bear on the problem with anything like adequate force. A comparatively small sum of money would be needed to make Cyprus a health resort with conspicuous advantages and to decrease enormously the "spleen rate" of its population. Sir Ronald's Report has been submitted to the Colonial Office, and there is every hope that shortly a more sustained and profitable attack will be made on the mosquito-breeding puddles in the island, and that British administration may be freed from the reproach attached to it as regards its treatment of this important possession, while such excellent results have been obtained in Ismailia, Havana, and other places.

### A MENTAL DEFICIENCY COMMITTEE FOR LONDON.

The General Purposes Committee of the London County Council, having had under consideration the Council's position under the Mental Deficiency Act, suggest that a special committee, to be known apparently as "The Asylums and Mental Deficiency Committee," shall be appointed. Exclusive of *ex-officio* members there are to be forty members, eight not being members of the County Council, and not fewer than four being women. The members from outside the Council are to be Poor-Law Guardians or other persons having special knowledge and experience with respect to the care, control, and treatment of defectives. The Council has applied to Parliament for legislation by which the new committee will also serve as the Visiting Committee for the purposes of the Lunacy Acts; but until the two services are thus co-ordinated it is proposed that the Council's medical officer of health shall be the officer responsible for the direction of the medical side of the mental deficiency service. The clerk of the Asylums Committee will act for the time being as the clerk of the new committee. At the Council's meeting last week, after much discussion, the matter was referred back to the General Purposes Committee.

### TWO EXPERIMENTERS' SUDDEN DEATH.

The deaths of two research workers abroad are recorded this week in the persons of Professor Joachimstal, director of the University Hospital for the Surgical Treatment of Cripples, Berlin, and of Dr. Fox, an Australian scientist, in Calcutta. The correspondent of the *Times* states that Professor Joachimstal died from inflammation of the lungs and "a nervous complaint which followed a disease of unknown character, which he contracted in the course of his experiments on animals." Physical malformations were his specialty, and it is reported that the Berlin medical men who attended him have been unable at present to determine the nature of the poison. There has been no room for doubt in the case of Dr. Fox, who was demonstrating his antidote for snake poison to the Indian authorities, but who unfortunately, we learn from Reuter's correspondent, overlooked one of five punctures which he received from a snake while demonstrating in the Zoo at Calcutta. The four punctures were apparently treated with success, but it was not till symptoms of poisoning manifested themselves that the fifth was discovered, with the result that it was too late to employ the antidote successfully, as it proves effective only when immediately applied. These two deaths, it can only be hoped, will compensate, in some degree, for the loss of two enthusiastic investigators by adding to our knowledge of the causes for which they worked.

### THE SALARIES OF POOR-LAW CLERKS.

Whilst congratulating Mr. F. W. Piper, clerk to the Wandsworth Board of Guardians, on his promotion to the position of superintendent registrar of births and deaths for Wandsworth, regret is expressed at the loss to the Poor-Law Service of so capable an official. Mr. Piper has been close on twenty-one years associated with the Wandsworth Board of Guardians, and for the last thirteen has acted as clerk. His salary was £500 a year, the limit allowed to a Metropolitan Poor-Law clerk by the Local Government Board. The attitude taken by the Local Government Board on this question is remarkable. They decline to increase the salaries of Metropolitan Poor-Law clerks beyond £500 a year, yet sanction much larger salaries for provincial Boards, and, as in a recent instance, approve of the appointment of officers to Unions outside London at enhanced salaries, who, a few months before, had been refused an increase of salary as clerks to a Metropolitan Board of Guardians. When, as in the case of Mr. Piper, officers take positions outside the Service at larger salaries, with less responsibilities, there is a distinct loss to the administration of the Poor Law in London.

### INTERESTING STATISTICS FROM THE LONDON HOSPITAL.

The London Hospital Committee presented an interesting report on Wednesday, from which we take the salient points. The Quinquennial Appeal resulted in £103,670 gross, with expenses £6,820. It is interesting to remember that the amount obtained from the Quinquennial Appeal of 1908 was £72,564. We note also that the success of last year's appeal is attributed to individual efforts rather than to general advertisement. The number of in-patients treated, viz. 17,096, constitutes a record, the highest number of previous years being 16,884 in 1911, and the mean residence of each is 17.78 days, a reduction attributed to the lessened shock which follows modern operations. Appendicitis patients leave in a fortnight instead of after a month as they did a few years ago. The total number of anæsthetics administered in 1913 was 22,080, and there were only seven cases of death. Although the number of out-patients has decreased to 173,774 in 1913, as compared with 220,729 in 1912, due in some degree to the Insurance Act, their character has somewhat changed, in that many more individuals are now sent to the Hospital by panel doctors for some special form of treatment or for a consultation. Moreover, statistics would seem to show that the number of out-patients bears a relation to trade and employment. Last year as regards trade was very favourable, and this may account in some measure for the decrease of patients.

### COMING NEW EXTENSIONS AT THE "LONDON."

The following developments at the London Hospital are contemplated: A Salvarsan Ward, Aural Wards, and a Hostel for Resident Doctors. Thanks to the Company of Grocers' offer of a sum up to £10,000 for the building, the special wards for the treatment of syphilis will consist of about fifteen separate rooms, with laboratories, etc., plans for which have recently been submitted to the Grocers' Company for consideration. The number of beds available for aural cases has hitherto been only twenty, which is an entirely inadequate number in proportion to the 9,883 cases which are treated as out-patients. Owing to the shortage of beds for this department, many patients have to be placed under the care of a general surgeon who would otherwise be treated by the aural surgeons. Plans have, therefore, been drawn out for the building of aural wards which will contain thirty beds. The progress of medicine in recent years has been more on the laboratory side than on the clinical side, which has rendered necessary increased laboratory accommodation in the hospital; it has, therefore, been decided to build a Hostel for Resident Doctors, which will have the double effect of placing in one building the various doctors who now have rooms scattered all over the hospital; and the rooms so freed will be available for the extension of the clinical laboratory, the cardiac, radiographic, and other departments. The block of houses opposite the out-patient department, between Pasteur Street and Mount Street, have been acquired as a site for this hostel. Extension will be necessary of the laundry, and also of the quarters for nurses.

### THIS WEEK'S DRUG MARKET.

Quieter conditions have prevailed during the week and transactions have been on a very limited scale. The cod-liver oil market continues to attract attention; the results of the fishing in Norway have been much better and prices have continued to tend downwards. There is very little change in the position of opium; the demand for morphine continues good, prices being unaltered; makers of codeine have reduced their quotations. Quillaia bark is in short supply and prices are firmly maintained. Cantharides are scarce and dearer. Eucalyptus oil is in very slow demand. Indian hemp tends dearer. Belladonna leaves, extract, and root are dearer. Quotations for strychnine have been reduced. Castor oil is rather dearer. Veratrine is lower in price. The price of citric acid has an upward tendency. The value of essence of lemon has again been raised, but the opinion seems to prevail that prices will decline again. American peppermint oil is dearer. There seems to be little prospect of lower prices for olive oil, the tendency being rather in the other direction. Menthol is very quiet.

